# Understanding Routine Treatment and Resource Availability for Fatigue in Multiple Sclerosis in the United Kingdom

**DOI:** 10.1155/bn/8169644

**Published:** 2026-03-09

**Authors:** Federica Picariello, Madison Milne-Ives, Jennifer Freeman, Rona Moss-Morris

**Affiliations:** ^1^ Health Psychology Section, Institute of Psychiatry, Psychology and Neuroscience, King′s College London, London, UK, kcl.ac.uk; ^2^ Faculty of Health, School of Health Professions, University of Plymouth, Plymouth, UK, plymouth.ac.uk

**Keywords:** fatigue, implementation, multiple sclerosis, routine care, UKMSR

## Abstract

**Background:**

Fifty‐five to ninety percent of people with multiple sclerosis (MS) report disabling fatigue, but a previous survey found that only 31% of UK adults with MS experiencing fatigue report being offered any treatment. The aim of this study was to capture the service perspective of routine care for MS fatigue and resource availability.

**Methods:**

A cross‐sectional online survey was distributed to NHS services across the United Kingdom to be completed by healthcare professionals working with people with MS.

**Results:**

Eighteen services completed the survey. Services supported a median of 1100 patients. Allied healthcare professional availability varied across services but was generally low (median range 0–6 staff per type). On average, services estimated that only 28% (*S* *D* = 26.51) of patients were receiving fatigue treatments.

**Conclusions:**

Findings highlighted the unmet need around MS fatigue management, complementing previously captured patient perspectives. Although the study included a small, self‐selecting subset of services and may have been influenced by the COVID‐19 pandemic, it demonstrates the necessity of accounting for resource availability to successfully implement new fatigue interventions.

## 1. Introduction

Fatigue, although experienced by many people with MS (pwMS) (55%–90%) and often debilitating, is undertreated [1–3]. Whilst UK National Institute for Health and Care Excellence guidelines recommend medication, exercise and behavioural treatments [4], medications show limited efficacy compared to nonpharmacological interventions [5, 6], and routine fatigue care is yet to be formalised [1]. Translating fatigue treatment research into practice is limited by evidence quality [7, 8], a lack of consideration of delivery context in treatment development and limited MS specialist healthcare professionals (HCPs) available to deliver treatments [9]. To address this research‐to‐practice gap, we need to understand current routine care. This study is aimed at (1) describing routine MS fatigue care in the UK National Health Service (NHS) and (2) determining the resources available to integrate fatigue treatments into routine care across the NHS from a service perspective. This complements our survey examining fatigue treatment from the patient perspective, where 22% of pwMS reported being offered medication, 6% behavioural therapy and 3% exercise [1].

## 2. Materials and Methods

A cross‐sectional online survey of NHS services providing care to pwMS (hospitals or community centres) was distributed through the UK MS Register (UKMSR) to all their 48 NHS partner sites, as well as via the MS Society′s network of services, professional conferences and personal channels. The UKMSR was approved as a study by the South West Central Bristol Research Ethics Council (initial: 11/SW/0160; renewed: 16/SW/0194) [10]. Study approval was obtained from the UKMSR internal ethical committee.

Data were collected using REDCap (March–July 2021). HCPs working with pwMS were invited to complete the questionnaire; this was broadly defined to include any relevant HCPs at the service (e.g. nurses, physiotherapists and neurology consultants) and could be completed collaboratively by a team. The questionnaire was developed with clinical stakeholders to capture service characteristics (including number of pwMS served, setting and specialisation), staff and resource availability and routinely offered fatigue treatments (Supporting Information). Data were stored and analysed using descriptive statistics in Excel.

## 3. Results

### 3.1. Service Characteristics

Eighteen responses were received from services across the United Kingdom (Greater London, South East, South West, West Midlands, East Anglia, North West, Wales and Northern Ireland). Most respondents were MS nurses/specialist nurses (7/19), but respondents also included occupational therapists (OTs) (4/19), neurologists (3/19), consultants (2/19), physiotherapists (2/19) and an MS practitioner (1/19); the survey was collaboratively completed by a consultant and physiotherapist at one service. Of the services represented, 67% (12/18) were outpatient based, 28% (5/18) were community based and one was inpatient based. Two‐thirds (12/18) were MS specific; the others were neurology. Services supported a median of 1100 pwMS (*I* *Q* *R* = 2090, range: 10–5000). Outpatient services tended to have larger populations (*M* *e* *d* = 2250, *I* *Q* *R* = 2050) than community services (*M* *e* *d* = 250, *I* *Q* *R* = 350).

### 3.2. Routine Fatigue Care

Fewer than half of services (8/18) reported routinely offering pharmacological treatments for fatigue. One hundred percent (8/8) offered amantadine and 38% (3/8) modafinil, but few patients at these services were prescribed fatigue medication (*M* *e* *d* = 15*%*, *I* *Q* *R* = 18.75). Most services reported routinely offering occupational therapy (13/18) or exercise (12/18) for fatigue; fewer offered psychological therapy (8/18) or social care assessments (2/18) for fatigue (Table 1).

**Table 1:** Subtypes, facilitators and settings of delivery of nonpharmacological treatments offered routinely for fatigue across services (*n* = 18; subtypes offered are given as a percentage of the number of sites offering some type of treatment in that category).

Category	Type	*N* (%)^a^
*Occupational therapy*		13 (72)
Facilitator	Specialist neuro‐occupational (MS) therapist	7 (54)
	General (non‐MS specialist) occupational therapist	7 (54)
	MS specialist nurse	3 (23)
	Other (*physiotherapist*)	1 (8)
	Psychologist	0 (0)
	Counsellor	0 (0)
Setting	Community	9 (69)
	Outpatient	5 (39)
	Inpatient	1 (8)
*Exercise*		12 (67)
Treatment subtype	General advice	11 (91)
	Balance	8 (67)
	Aerobic	6 (50)
	Resistance	6 (50)
	Vestibular rehabilitation	5 (42)
	Yoga	4 (33)
	Referral to GP or community	4 (33)
	Other (Pilates, individualised exercise plan, exercise as part of general fatigue management course)	4 (33)
Facilitator	Specialist neurophysiotherapist (MS)	9 (75)
	Specialist neuro‐occupational (MS) therapist	8 (67)
	MS specialist nurse	5 (42)
	Referral to community teams	4 (33)
	Psychologist	3 (25)
	General (non‐MS specialist) occupational therapist	3 (25)
	Referral to specialist rehabilitation unit	2 (17)
	Dietitian	2 (17)
	Other (*GP*, *consultant*)	2 (17)
	General (non‐MS specialist) physiotherapist	1 (8)
Setting	Community	9 (75)
	Outpatient	8 (67)
	Inpatient	1 (8)
*Psychological therapy*		8 (44)
Treatment subtype	FACETS (Fatigue: Applying Cognitive behavioural and Energy effectiveness Techniques to LifeStyle; 6‐week group programme)	8 (100)
	Mindfulness	3 (38)
	Counselling	2 (25)
	Cognitive behavioural therapy (CBT)	2 (25)
	Other (motivational interviewing)	1 (13)
Facilitator	Specialist neuro‐occupational (MS) therapist	7 (88)
	MS specialist nurse	3 (38)
	Psychologist	2 (25)
	General (non‐MS specialist) occupational therapist	2 (25)
	Other (*physiotherapist*)	1 (13)
	Counsellor	0 (0)
Setting	Outpatient	6 (75)
	Community	5 (63)
	Inpatient	1 (13)
*Social care assessment for fatigue*		2 (11)
Facilitator	Social carer	2 (100)
	Other (specialist neuro‐occupational therapist)	1 (50)
Setting	Community	2 (100)
	Outpatient	0 (0)
	Inpatient	0 (0)

^a^Percentages exceed 100% as multiple responses are allowed.

Services estimated that a mean of 28% of pwMS (*S* *D* = 26.51; *M* *e* *d* = 20, *I* *Q* *R* = 23.75) were receiving fatigue treatment and HCP respondents did not feel that fatigue management needs were being well met (mean rating = 33.94/100, *S* *D* = 21.04; *M* *e* *d* = 30, *I* *Q* *R* = 33.5), with one respondent commenting that ‘*fatigue remains one of the symptoms less well treated*’. Lack of availability or resources was the most common reason given for the rating, with respondents highlighting issues such as the ‘*lack of services in our area means unable to refer onto more specialist [services] with fatigue management’*, and ‘*the service is very good, but the waiting time is long because there just aren′t enough therapists*’.

### 3.3. Availability and Access to MS Nurses and Allied HCPs

Types of staff available varied across services, with general (non‐MS specialist) and specialist neurophysiotherapists (MS) and MS specialist nurses most common (Table 2). Access to other types of staff was more restricted, particularly counsellors and social workers. General physiotherapists, dietitians, counsellors and social workers were more frequently available through community care, whilst MS nurses, specialist physiotherapists, general OTs and psychologists were more available through outpatient care. Estimated percentages of pwMS with access to various HCPs (given that that HCP type was available in the service) ranged from 63% to 88%. Services accessing resources through linked community or general hospital services described the referral process as easy but highlighted that ‘*waiting lists often cause delays in care’*. Provision was also described as a ‘*postcode lottery*’ and not universally accessible due to ‘*distance and transport*’.

**Table 2:** Overview of MS nurses and allied healthcare professionals (HCPs) available and accessible across services surveyed (*n* = 18).

HCP type	No. of services with at least one of the HCP type in their service, *N* (%)	Median number of HCPs per service (*n* = 18), Med (IQR; range)	Inpatient based, *N* (%)^a^	Outpatient based, *N* (%)^a^	Community based, *N* (%)^a^	% of pwMS estimated to have access to HCP where available, M (SD)
MS nurse	18 (100)	3 (2.75; 1–11)	10 (56)	13 (72)	10 (56)	88 (19.72)
Specialist neurophysiotherapist (MS)	14 (78)	3 (4; 0–40)	7 (50)	10 (71)	8 (57)	74 (30.77)
General physiotherapist	12 (67)	6 (10; 0–60)	7 (58)	7 (58)	10 (83)	77 (28.59)
Specialist neuro‐occupational (MS) therapist	9 (50)	1 (4.75; 0–20)	7 (78)	4 (44)	5 (56)	82 (30.02)
General occupational therapist	10 (56)	3 (5; 0–20)	7 (70)	7 (70)	7 (70)	78 (29.20)
Dietitian	10 (56)	2 (2.75; 0–10)	6 (60)	5 (50)	8 (80)	70 (37.72)
Psychologist	10 (56)	1 (2; 0–9)	4 (40)	7 (70)	4 (40)	63 (43.48)
Counsellor	4 (22)	0 (0; 0–10)	1 (25)	1 (25)	4 (100)	67 (38.43)
Social worker	7 (39)	0 (8.75; 0–20)	5 (71)	2 (29)	7 (100)	66 (40.27)
Other (speech and language therapists, specialist neuropharmacists, podiatrists, neurorehab consultants and continence teams; excluding neurologists)	10 (56)	—	2 (20)	7 (70)	7 (70)	73 (38.63)
Community rehabilitation services	15 (83)	—	—	—	—	68 (37.83)

Abbreviations: HCP, healthcare professional; IQR, interquartile range.

^a^Inpatient, outpatient, and community‐based HCP percentages exceed 100% as some services had access through multiple pathways, which reflects the percentage of the total number of sites where that HCP was available.

## 4. Discussion

Reports from 18 NHS services highlighted a clear insufficiency of resources and access to MS fatigue treatments. Other than MS nurses, allied HCPs who may be able to offer fatigue treatments were not consistently accessible. MS nurses already have caseloads ~1.5 times the recommended number (472 vs. 315 pwMS) [9]; adding fatigue specialist treatment to this workload, which involves overall care and disease‐modifying treatments, would be challenging. These insights on resource availability need to guide fatigue treatment development to ensure their implementability in routine care. For instance, cognitive behavioural therapy for fatigue delivered by psychologists will be challenging, as many services have limited or no access to psychologists.

There were some discrepancies between staff and patient reports of accessibility to fatigue treatments. In our patient‐facing survey, less than a third of patients reported being offered treatment (22% medication, 6% behavioural therapy, 3% exercise and 3% OT) [1]. This contrasts with service reports that exercise and OT were offered routinely at more sites than pharmacological and psychological treatments. A response option for the question about exercise treatments (‘general advice regarding physical activity’) was not in the patient‐facing survey. Over 60% of services (11/18) reported offering general advice, whilst no more than one‐third (6/18) offered any of the other exercise treatment types, which may explain this difference. However, both pwMS and services reported the need for better fatigue treatment provision, with similar estimates of fatigue treatment receipt (pwMS: 31% [1], services: 28%).

Limitations of the study were the relatively small sample size—likely influenced by reduced capacity to respond during the COVID‐19 pandemic—and lack of triangulation with objective data. As the study took place during a period of disruption to healthcare services, resource availability and estimates of how many pwMS would have access to certain staff and treatments may also have been affected by pandemic‐related strain. To mitigate this, the survey requested that responses be based on staffing and resources available before the pandemic started. There is also potential selection bias from the services that chose and had the capacity to complete the survey, although any selection bias is likely to be towards better resourced services that had the capacity to complete the survey, potentially masking weaker fatigue care provision. For anonymity, to help ensure accurate depictions of care provision, we did not require respondents to name their NHS service. This means we cannot provide response rates; however, from the data captured on region, setting (inpatient, outpatient or community), level of specialisation and approximate number of pwMS served, no duplicate service responses were detected.

## 5. Conclusions

Despite the prevalence and significant impact of fatigue for pwMS, surveys of patients [1] and NHS services highlighted a substantial unmet need for fatigue management. Better understanding of the context of fatigue treatment delivery and access pathways will support integration of new treatments in routine care, help realise patient benefits [1] and could inform national guidelines [8]. Inconsistency in resources across services is a critical challenge for treatment implementation and necessitates the capacity for flexibility in delivery. A one‐size‐fits‐all approach to MS fatigue treatment is unlikely to be successful.

## Author Contributions

**F.P.**: conceptualisation, methodology, investigation, formal analysis, writing—original draft and funding acquisition; **M.M-I.**: formal analysis and writing—review and editing; **J.F.**: conceptualisation, methodology and writing—review and editing; **R.M-M.**: conceptualisation, methodology, writing—review and editing, supervision and funding acquisition.

## Funding

R.M‐M. acknowledges the financial support from the Department of Health via the National Institute for Health Research (NIHR) Specialist Biomedical Research Centre for Mental Health award to the South London and Maudsley NHS Foundation Trust (SLaM) and the Institute of Psychiatry at King′s College London.

## Disclosure

The views expressed in this article are those of the authors and not necessarily those of the NHS, the NIHR, or the Department of Health. The funders had no role in the study design, data collection, data analysis, data interpretation, or writing of the report. The authors had access to all study data and final responsibility for the decision to submit for publication.

## Conflicts of Interest

The authors declare no conflicts of interest.

## Supporting information


**Supporting Information** Additional supporting information can be found online in the Supporting Information section. Table S1: An overview of the data requested in the questionnaire sent to MS services.

## Data Availability

Data cannot be made publicly available as it is held by the UKMSR and falls under their privacy protocols.

## References

[1] Picariello F. , Freeman J. , and Moss-Morris R. , Defining Routine Fatigue Care in Multiple Sclerosis in the United Kingdom: What Treatments Are Offered and Who Gets Them?, Multiple Sclerosis Journal–Experimental, Translational and Clinical. (2022) 8, no. 1, 20552173211072274, 10.1177/20552173211072274. 35096412 PMC8796089

[2] Young C. A. , Mills R. , Rog D. , Sharrack B. , Majeed T. , Constantinescu C. S. , Kalra S. , Harrower T. , Santander H. , Courtald G. , and Ford H. L. , Quality of Life in Multiple Sclerosis Is Dominated by Fatigue, Disability and Self-Efficacy, Journal of the Neurological Sciences. (2021) 426, 117437, 10.1016/j.jns.2021.117437. 33991718

[3] Moore H. , Nair K. P. S. , Baster K. , Middleton R. , Paling D. , and Sharrack B. , Fatigue in Multiple Sclerosis: A UK MS-Register Based Study, Multiple Sclerosis and Related Disorders. (2022) 64, 103954, 10.1016/j.msard.2022.103954. 35716477

[4] National Institute for Health and Care Excellence , Multiple Sclerosis in Adults: Management, 2022, National Institute for Health and Care Excellence.

[5] Cruccioli M. , Teixeira P. , Pitanga J. , da Silva L. T. , Rossi Y. I. , Koppanatham A. , Fraiman P. , and Sarmento F. , Amantadine for Multiple Sclerosis-Related Fatigue: A Systematic Review and Meta-Analysis of Randomized Controlled Trials (P7-1.002), Neurology. (2025) 104, 10.1212/WNL.0000000000208714. PMC13596907 41446963

[6] Moss-Morris R. , Harrison A. M. , Safari R. , Norton S. , van der Linden M. L. , Picariello F. , Thomas S. , White C. , and Mercer T. , Which Behavioural and Exercise Interventions Targeting Fatigue Show the Most Promise in Multiple Sclerosis? A Systematic Review With Narrative Synthesis and Meta-Analysis, Behaviour Research and Therapy. (2021) 137, 103464, 10.1016/j.brat.2019.103464. 31780252

[7] Harrison A. M. , Safari R. , Mercer T. , Picariello F. , van der Linden M. , White C. , Moss-Morris R. , and Norton S. , Which Exercise and Behavioural Interventions Show Most Promise for Treating Fatigue in Multiple Sclerosis? A network meta-analysis, Multiple Sclerosis Journal. (2021) 27, no. 11, 1657–1678, 10.1177/1352458521996002, 33876986. 33876986 PMC8474304

[8] Mulligan K. , Harris K. , Rixon L. , and Burls A. , A Systematic Mapping Review of Clinical Guidelines for the Management of Fatigue in Long-Term Physical Health Conditions, Disability and Rehabilitation. (2025) 47, no. 3, 531–548, 10.1080/09638288.2024.2353855, 38832888. 38832888

[9] Roberts M. , Chico D. , and Naik P. , Multiple Sclerosis Specialist Nursing in the UK 2021: Results From the MS Trust Nurse Mapping Survey, 2021, Multiple Sclerosis Trust.

[10] Middleton R. M. , Rodgers W. J. , Chataway J. , Schmierer K. , Rog D. , Galea I. , Akbari A. , Tuite-Dalton K. , Lockhart-Jones H. , Griffiths D. , Noble D. G. , Jones K. H. , al-Din A. , Craner M. , Evangelou N. , Harman P. , Harrower T. , Hobart J. , Husseyin H. , Kasti M. , Kipps C. , McDonnell G. , Owen C. , Pearson O. , Rashid W. , Wilson H. , and Ford D. V. , Validating the Portal Population of the United Kingdom Multiple Sclerosis Register, Multiple Sclerosis and Related Disorders. (2018) 24, 3–10, 10.1016/j.msard.2018.05.015, 2-s2.0-85047736520, 29860199. 29860199

