# Supplementary material for: Understanding Routine Treatment and Resource Availability for Fatigue in Multiple Sclerosis in the United Kingdom
Source: Behav Neurol. 2026 Mar 9;2026:8169644. doi: 10.1155/bn/8169644 (PMC13580807; doi:10.1155/bn/8169644)
Supplement: Supplementary file 1 [file BN-2026-8169644-s001.docx]

**Supplementary Material:** Overview of content covered in the questionnaire to services.

| Items | Responding format |
| --- | --- |
| Service characteristics | |
| Setting | - Inpatient - Outpatient - Community |
| Specialisation of the team | - MS - Neuro - Generalist team |
| Approximate number of pwMS served |  |
| Access to resources/staff | |
| - MS Nurses - Specialist Neuro (MS) Physiotherapists - Physiotherapists (general) - Specialist Neuro (MS) Occupational Therapists - Occupational Therapists (general) - Dietitians - Psychologists - Counsellors - Social Workers - Community Rehab Services - Any other healthcare professionals | - Inpatient - Outpatient - Community - None   Respondents were also asked to provide: (1) an estimate of numbers of each role in the service and (2) an estimate of the percentage of pwMS who likely get access (0-100%). |
| Comments in relation to referral process and waiting-list if access to certain treatments through linked community or general hospital services | Free text response |
| Routine fatigue treatments | |
| Medications for fatigue | No  Yes:   - Amantadine (Symmetrel or Lysovir) - Modafinil (Provigil) - Prokarin (a skin patch that contains caffeine and histamine) - Other prescription medications [free text box] - Over the counter medications/supplements (such as liquid iron, beetroot powder, vitamin B12)   An estimate of the percentage of pwMS who would be offered any of these medications (0-100%) |
| Exercise | No  Yes:   - Aerobic (exercise that causes you to be quickly out of breath, like running, swimming, walking, hiking) - Resistance (exercise that improves muscular strength and endurance, like squats, bicep curls) - Yoga - Balance (exercises that improve your ability to control and stabilize your body's position) - Vestibular rehabilitation - Referral to GP for exercise on prescription / exercise referral scheme at local leisure centre - General advice regarding physical activity and fitness - Other exercise [free text box]   Respondents were asked to indicate where this would be offered (Inpatient, Outpatient, Community) and by whom (Specialist Neuro (MS) Physiotherapist, Physiotherapist (general), Specialist Neuro (MS) Occupational Therapist, Occupational Therapist (general), Referral to specialist rehab units, Referral to community teams, MS Nurse, Psychologist, Dietitian, Other [free text]) |
| Behavioural or psychological management | No  Yes:   - Counselling - Cognitive-behavioural therapy (CBT) - Mindfulness - FACETS (six group sessions focused on self-management of fatigue) - Other psychological approach [free text box]   Respondents were asked to indicate where this would be offered (Inpatient, Outpatient, Community) and by whom (MS Nurse, Psychologist, Counsellor, Occupational Therapist (general), Specialist Neuro (MS) Occupational Therapist, Other [free text]) |
| Social care assessment (support with tasks social services can help with) | No  Yes  Respondents were asked to indicate where this would be offered (Inpatient, Outpatient, Community) and by whom (Social Worker, Other [free text]) |
| Occupational therapy (including support with energy-saving gadgets at work or around the home) | No  Yes  Respondents were asked to indicate where this would be offered (Inpatient, Outpatient, Community) and by whom (Specialist Neuro (MS) Occupational Therapist, Occupational Therapist (general), MS Nurse, Psychologist, Counsellor, Other [free text]) |
| Any other treatments | No  Yes [free text]  Respondents were asked to indicate where this would be offered (Inpatient, Outpatient, Community) and by whom (Neurologist, MS Nurse, Specialist Neuro (MS) Physiotherapist, Physiotherapist (General), Specialist Neuro (MS) Occupational Therapist, Occupational Therapist (general), Psychologist, Counsellor, Dietitian, Social Worker, Referral to specialist rehab units, Referral to community teams, Other [free text]) |
| Availability of individual in service who leads/enables the provision of MS fatigue treatments | No  Yes [free text] |
| An estimate of percentage of pwMS who receive of these fatigue treatments | 0-100% |
| Rating of extent available services for fatigue meet pwMS’ needs | On a scale from 0 (Not at all meeting pwMS’ needs) and 100 (Extremely well meeting pwMS’ needs)  Respondents were asked to briefly explain the rating [free text] |
